# Patients’ expectations of variation in quality of care relates to their search for comparative performance information

**DOI:** 10.1186/s12913-014-0617-y

**Published:** 2014-12-03

**Authors:** Nicole ABM Ketelaar, Marjan J Faber, Jozé C Braspenning, Gert P Westert

**Affiliations:** Scientific Institute for Quality of Healthcare (114), Radboud University Medical Center, PO Box 9101, 6500 HB Nijmegen, The Netherlands

**Keywords:** Expectations, Comparative performance information, Practice variation, Quality of care, Hip replacement, Knee replacement, Elective surgery

## Abstract

**Background:**

Choice of hospital based on comparative performance information (CPI) was introduced for Dutch healthcare consumers at least 5 years ago, but CPI use has not yet become commonplace. Our aim was to assess the role of patients’ expectations regarding variation in the quality of hospital care in determining whether they search for CPI.

**Methods:**

A questionnaire (for a cross-sectional survey) was distributed to 475 orthopaedic patients in a consecutive sample, who underwent primary hip or knee replacement in a university, teaching, or community hospital between September 2009 and July 2010.

**Results:**

Of the 302 patients (63%) who responded, 13% reported searching for CPI to help them choose a hospital. People who expected quality differences between hospitals (67%) were more likely to search for CPI (OR =3.18 [95% CI: 1.02–9.89]; *p* <0.04) than those who did not. Quality differences were most often expected in hospital reputation, distance, and accessibility. Patients who did not search for CPI stated that they felt no need for this type of information.

**Conclusion:**

Patients’ expectations regarding variation in quality of care are positively related to their reported search for CPI. To increase the relevance of CPI for patients, future studies should explore the underlying reasoning of patients about meaningful quality-of-care variation between hospitals.

## Background

The public release of comparative performance information (CPI) is common in many countries [1]. The 2006 Healthcare Market Regulation Act led to better availability of CPI in the Netherlands. Choice for consumers and providers became a cornerstone of this new healthcare system based on market elements and competition. CPI can include information about service, patient experiences, and quality indicators for clinical care (structure, process, and patient outcomes). The CPI in the Netherlands includes items related to the hospital (e.g. ranking of 100 hospitals) and to condition-related factors (e.g. patient experiences, waiting lists, and annual patient volumes). The purpose of making this information publicly available is to enable healthcare consumers to choose high-quality healthcare [2] and to empower them to make an informed choice about healthcare [3].

There is no firm evidence that CPI influences patient choices [4]. American and British studies have shown that the actual use of CPI for hospital care is restricted to 4–14% of the consumers [5,6], while the idea of choice appeals to most consumers [7]. Several Dutch studies of CPI for total hip or knee replacements have been performed [8-11]. The patients in Moser and colleagues’ study considered CPI to be an additional source of information when they were preparing for a doctor’s appointment. They benefited from the information most when they had to undergo a total hip or knee replacement for the first time [9]. American patients who report a lack of hospital choice for total hip or knee replacement are more likely to be dissatisfied with their surgery [8]. This observation suggests that encouraging patients to engage in provider selection based on quality of care would improve their satisfaction.

The low level of CPI search behaviour for selecting a hospital is partly due to the consumer’s previous experience with a specific hospital [12-14] and the unfamiliarity of using CPI for hospital selection [15,16], as well as the role of the referring physician. Dutch research shows that many patients prefer their primary care physician to be involved in the choice of hospital so they can either take the physician’s advice or delegate the decision [17-19]. Furthermore, many consumers are unaware of the opportunity to consult CPI [6,20,21].

To raise the level of CPI awareness (an important step in a causal chain towards using CPI) an attendant motive is required so that consumers feel a need for this information [21]. One consumer choice model takes awareness as a starting point [18]. Consumers must be aware that there is CPI, and that it is possible to make a choice. We presume that patients’ expectations are an important pre-step and have a subjective influence on awareness. Studies show that, when consumers start searching for information, the questions and expectations already in their minds will drive the direction of their search [22-24]. Having expectations about the practice variation of hospitals and therefore perceiving a risk of receiving poor care might be a motive for using CPI.

Despite on-going efforts and investments in the collection, production, and dissemination of CPI for the public [15], there is no firm evidence that CPI influences patient choices [4]. In an attempt to bridge this gap, we hypothesise that consumers who expect to find quality differences between hospitals are those who search for CPI. We tested whether patients’ expectations of variation in quality of care affect their reported search behaviour for hospital performance information, then we adjusted for potential confounders. Furthermore, respondents were asked in what ways they expected hospitals to differ. We also asked them about the most important reasons why they did not search for CPI. We obtained data from patients who had recently been admitted to hospital for an elective total hip or knee replacement. Admission to a hospital for elective surgery can be planned in advance, which gives patients time to search for and look into CPI. These replacement procedures are provided at all 87 hospitals in the Netherlands.

## Methods

In a cross-sectional study of three types of hospitals, we used a consecutive sampling strategy to recruit 475 patients undergoing a total hip or knee replacement, and we invited them to participate in a paper-based survey. To make our sample representative, we included patients from a university hospital, a teaching hospital, and a community hospital. We included adult patients undergoing a *primary* hip or knee replacement because we expected that previous surgical experience (as in the case of a secondary replacement) would bias the selection. The annual patient volumes were 53 for primary hip replacements and 64 for knee replacements in the university hospital; 232 and 170, respectively, in the teaching hospital; and 236 and 153, respectively, in the community hospital. Data collection took from 5 to 9 months (September 2009 through July 2010). The nurse or anaesthetist who prepared the patient for surgery personally gave the survey questionnaire to the patient at the preoperative appointment 4 weeks prior to the operation. Reminders were sent 2–3 weeks later in the teaching and community hospitals, but no reminders were sent in the university hospital because permission for this was not granted.

**Table 1:** **Characteristics of the participants differentiated by type of hospital** ^**†**^

	**Total**		**University hospital**		**Teaching hospital**		**Community hospital**	
	***n***	**%**	***n***	**%**	***N***	**%**	***n***	**%**
**Women**	158	57	45	55	65	57	48	58
**Men**	121	43	37	45	49	43	35	42
Age ≤65*	107	40	45	56	42	39	20	25
>65*	163	60	35	44	67	62	61	75
**Low level of education**	222	81	64	79	86	76	72	90
**High level of education**	52	19	17	21	27	24	8	10
**Total primary** ***hip*** replacements*	179	65	58	72	69	61	52	63
**Total primary** ***knee*** **replacements**	97	35	23	28	44	39	30	37
**Received** ***no*** **previous treatment in current hospital**	59	21	27	34	18	16	14	17
**Received previous treatment in current hospital**	217	79	53	66	98	84	68	83
**Did** ***not*** **receive hospital choice options from GPs**	217	80	58	72	94	84	65	82
***Received*** **hospital choice options from GPs**	55	20	23	28	18	16	14	18
***Unaware*** **that they could compare hospital performance**	67	24	22	27	26	23	19	23
***Aware*** **that they could compare hospital performance**	210	76	60	73	87	77	69	77
**Did** ***not*** **search for CPI**	242	87	68	83	97	85	77	93
***Searched*** **for CPI**	37	13	14	17	17	15	6	7
**Expectations of quality differences in hospital care**								
**No differences**	91	33	17	21	36	32	38	46
**Yes, small differences**	137	49	37	45	59	52	41	49
**Yes, large differences**	51	18	28	34	19	17	4	5

*Not every score accumulates to 279 because of missing characteristic data.

^†^Data were based on answers from eligible respondents about the search for comparative performance information and expectations of quality differences in hospital care.

CPI = comparative performance information; GP = general practitioner.

The institutional ethics committee reviewed the study protocol in accordance with local regulations in the Netherlands, and they concluded that the study was not subject to the Dutch Medical Research Involving Human Subjects Act.

The primary outcome measure was the self-reported search for CPI, i.e. ‘*Did you search for additional CPI to compare hospitals after it became clear that you needed surgery?’* (answer: yes or no). Respondents did not search for CPI were asked to select their motive from a list of seven pre-listed reasons with an option to add one. The reasons were based on a literature search (unpublished search) and focus group interviews with consumers [25]. Other measures concerned the previous treatment in the selected hospital: consumers’ perception of being well-informed to make a decision, the general practitioner’s (GP’s) role in advising a choice of hospital (all dichotomous variables), and expectations of variation in quality of care between hospitals (large, small, or no difference in quality of care). If respondents expected quality-of-care differences, they were asked to specify these differences for 14 pre-listed factors that were available from the Dutch internet sites KiesBeter.nl (‘Choose Better’) and Independer.nl. Both sites based the factors they listed on a set of quality indicators that the Health Care Transparency Programme listed in 2009 [26]. Focus group interviews with consumers [25] and the annual list of a Dutch magazine, Elsevier [27], also presented factors to be considered. The factors included the available CPI for hip and knee replacements in the Netherlands, which gave the name of the organisation and the clinical performance of the care providers. Formally, ‘distance’ and ‘reputation’ are not CPI, but they were included because they are important to patients [9,11,28]. In this sense, such information can be seen as part of the performance of the healthcare system. We used a broad definition of performance, as did Van Loon and Tolboom, who defined three information types: (1) factual information (names, addresses, and type of provider), (2) quality information based on performance, and (3) quality information based on consumer experience [29]. The questionnaire contained items about demographic variables (age, education, and type of replacement [hip or knee]), and hospital characteristics (type and patient volumes for hip and knee replacements).

We used descriptive statistics and frequency tables to describe our study population’s demographic variables (age, gender, and education), previous treatment in the current hospital, awareness that hospital performance information is available for comparison, receipt of choice options from GPs, the search for CPI, and expectations of quality differences in hospital care (Table 1). To address the issue of representativeness, we compared the characteristics of the participants in our study with the characteristics of a larger sample of 1508 Dutch patients who underwent a hip or knee replacement [30].

We included data only for those respondents who provided valid answers for the core items of our study: search for CPI and expectations of quality differences in hospital care. The respondents were dichotomised into a group 65 years or younger, and a group older than 65 years. The education variable (the level of education) was measured on a four-point scale (none, low, middle, and high), and it was dichotomised for analytical purposes into low and high levels of education.

We used logistic regression to analyse the relationship between expectations of quality differences (independent variable) and searching for CPI (dependent variable) for patients who underwent a hip or knee replacement (Table 2, model 1). In order to correct for possible confounders, we also performed this univariate analysis for the demographic variables (e.g. age, gender, education, and type of replacement), hospital type, GPs’ role in advising a choice of hospital, awareness of available information for comparing hospital performance, and previous treatment at the current hospital. Potential confounders (*p* <0.2) in univariate analyses were added to the multivariate model, and we examined their effects on the beta coefficients. Any variables resulting in a change in the beta coefficient of more than 10% were included in the final model. We compared the univariate analysis (model 1) with the multivariate analysis (model 2) for odds ratios (ORs) with 95% confidence intervals (95% CIs). An association was considered statistically significant for *p* <0.05.

**Table 2:** **The relationship between quality expectations and searching for comparative performance information by patients who underwent a hip or knee replacement (model 1), controlled for patient and hospital characteristics and previous treatment (model 2)**

	**Model 1** ^**†**^		**Model 2** ^**$**^	
	**Univariate analysis**		**Multivariate analysis**	
	**OR [95% CI]**	***p***	**OR [95% CI]**	***p***
Age				
<65			1.18 [0.55–2.53]	0.66
≥65 (reference)				
Hospital type				
Teaching			1.27 [0.54–2.95]	0.58
Community			0.73 [0.24–2.22]	0.58
University (reference)				
Previous treatment in current hospital				
Yes			0.32 [0.15–0.70]	0.00*
No (reference)				
Expectations regarding quality differences in hospital care		0.00*		0.04*
Yes, small differences	3.71 [1.22-11.27]	0.02*	3.18 [1.02–9.89]	0.04*
Yes, large differences	7.44 [2.28-24.30]	0.00*	5.05 [1.44–17.77]	0.01*
No, differences (reference)				

*P <0.05; OR [95% CI] = odds ratio [95% confidence interval].

^†^Based on the answers of 279 respondents.

^$^Based on the answers of 263 respondents.

We present descriptive statistics for the factors that we expected to differ between hospitals, as well as statistics for the reasons for not searching CPI. In describing these reasons, we distinguished between respondents who expected differences in the quality of hospitals and those who did not. We used SPSS 18.0 for all analyses.

## Results and discussion

### Study population

Of the 475 questionnaires sent out, 302 were returned completed (response rate 63%). 279 questionnaires had valid answers for the core items: search for CPI and expectations of quality differences in hospital care. Table 1 shows a comparison of consumer characteristics differentiated by the type of hospital. We compared our respondents with a sample of 1508 Dutch patients who underwent a hip or knee replacement [30]. The age distribution of our respondents was 40% for those aged 65 years or less and 60% for those older than 65 years. For the sample of 1508 patients, the age distribution was 30% and 70%, respectively. While this was more or less similar, the samples showed greater differences for gender: 43% were male in our study versus 28% in the sample of 1508 patients; and for a low level education, 19% and 28%, respectively.

### Awareness of hospital comparison information and choice

Most respondents (76%) reported that they were aware of the possibility of comparing hospital quality, and they (72%) were aware that they actually could choose a hospital for their surgery. Most respondents (73%) reported that they had a choice option. Most (89%) reported being well-informed about the choice of hospital. Of the total group of respondents, only 13% said they had searched for hospital CPI before choosing a hospital for the operation. Among those who did not have a choice option, a minority (14%) searched for information once they knew they needed surgery. It did not seem to matter whether patients had a choice option or not; a minority in each group searched for information.

### Expectations of differences in quality of hospital care

Most participants (67%) expected to find differences in hospital quality of care. The variables gender, type of replacement, GP role in advising a choice of hospital, and being aware that they could compare hospital performance contributed to the explanation model with *p* <0.2. However, these variables did not effect a substantial change (>10%) in the beta coefficients, so were excluded from the final model. Table 2 shows the results of the univariate and multivariate logistic regression analyses for the relationship between the quality expectations of hospital care and the reported search for CPI. Previous treatment in the current hospital also appeared to significantly influence the reported search for CPI.

The univariate model shows that respondents who expected small differences in hospital quality were more likely to search for CPI than those who did not (OR = 3.71 [95% CI: 1.22–11.27]). For people who expected large quality-of-care differences, this effect was even greater (OR = 7.44 [95% CI: [2.28–24.3]). The adjusted ORs were 3.18 [95% CI: 1.02–9.89] for the group who expected small differences, and 5.05 [95% CI: 1.44–17.77] for the group who expected large differences. Respondents with previous treatment in the same hospital less often searched for CPI than those who had no previous treatment (OR = 0.32 [95% CI: 0.15–0.70]).

The participants’ rating of expected hospital quality differences in reputation, distance, and accessibility are ranked the highest (Table 3).

**Table 3:** **Factors influencing choice of hospital for total hip and knee replacements** ^**†**^

**Hospital-related factors**	***n***
1. Reputation	107
2. Distance	95
3. Accessibility	79
4. Type of hospital (university, teaching, or community)	51
5. Ranking list of ‘100 hospitals’	41
6. Hospital size	20
7. Number of cancelled operations	1
**Condition-related factors**	***n***
8. Plan for pre-operative schedules on 1 day	51
9. Orthopaedic specialism	50
10. Patient experiences	49
11. PROMs	49
12. Waiting list	45
13. Annual patient volume	42
14. Infection rates	16

PROMs = Patient-reported outcome measures.

^†^Data were based on 191 answers from eligible respondents.

### Reasons for not searching for comparative performance information

Of the respondents who did not search for additional information, 179 said they felt no need for more information. Others gave far less common reasons: 19 had no internet access at home, 15 felt more information would create more doubts, 13 said searching for information was an extra burden, 7 thought choosing a hospital based on CPI was too much responsibility, 8 did not know where to look, 6 had no skills how to look, 8 had no hospital choice options, and 5 had no time.

Feeling no need was by far the most important reason for not searching for hospital CPI. Though, this reason was not significant related to the expectations of quality-of-care differences.

### Discussion

Thirteen per cent of our study population searched for CPI to compare hospitals. This is consistent with the results of American and Dutch studies for similar populations [14,31]. Our hypothesis that patients who expect quality differences are those who search for CPI was confirmed. Previous experience with the hospital is another factor influencing the search for CPI. Expecting quality of care differences in hospital performance appears to be a stimulus for searching for CPI, although respondents who underwent previous treatment in the hospital tended to search less for this information.

In our study, the impact of previous experience on hospital choice was consistent with Dixon and colleagues’ results [32]. They compared the effect of consumer choice during the referral process in the Netherlands and England. For Dutch patients, ‘being in the neighbourhood’ or ‘having been there before’ were the most important reasons for choosing a hospital: patients usually returned to the same hospital [33]. Interestingly, patients prefer being treated at their current hospital, even if they could choose a better alternative with higher-quality care [34,35]. Choosing a familiar hospital instead of an unknown one suggests that personal experience is a value in itself. While CPI may indicate the best hospitals, patients may optimise the factors they value most rather than objectively maximise quality.

Our respondents expected most differences to be in reputation, distance, and accessibility. As reported in other studies [6,12,19], these are known choice factors in decision-making. Our study population expected differences mainly in the general performance of the healthcare system rather than in specific condition-related factors (total hip or knee replacement). The factors for which consumers expect quality differences may change in the future as consumers become more knowledgeable about CPI. Dutch studies among patients with a total hip or knee replacement found that both interpersonal aspects (conduct of doctors) and more technical ones (for example, the prevention of adverse effects of thrombosis and the specialist area of orthopaedists) are important to patients [11]. Making the concept of quality more meaningful may also increase consumer interest and need for CPI [15]. Bozic and colleagues have confirmed this statement: they have recently found that patients were very motivated to search for provider quality. In their study, physician manner and surgical outcomes appeared to be the most important considerations for selecting a provider for elective total joint arthroplasty [36].

Some studies [6,18] led us to expect that unawareness is an important reason why hospital performance information has little influence on decision-making. An information need and a sense of urgency are necessary ingredients for awareness and interest in this type of information. Although most of our respondents (76%) were aware that they could compare hospital performance and choose a hospital (72%), they still did not use CPI. This discrepancy might be due to their feeling no need for such information. Having alternative information sources [9] or doubt about the trustworthiness of such information [2] could also contribute to this feeling. This discrepancy also implies the need for a more outspoken reporting of hospital quality that emphasises differences in quality. If rational patients assume that these differences are small, then they cannot be expected to look for and use such information. The on-going efforts and investments that go into the collection, production, and dissemination of CPI would then be useless.

### Implications

Our study shows that merely making CPI available in the public domain does not result in its use. This implies that further action, such as applying an implementation strategy, is necessary. Other studies see the need for an infrastructure that provides patients with advice about their choices and helps them in actually choosing [37,38].

Future research should explore the concept of patients’ expectations more comprehensively because the fact that patients’ expectations that quality differences exist affects their search for CPI, but does not affects their need for CPI. Research should use the resulting information to determine how fragile or robust these expectations are [39]. Whereas CPI is based on measurable factors, consumer expectations are more diffuse and individually determined. More development of tailored methods to assess the understanding of variation in quality of care as a precondition for acquiring awareness, knowledge, and interest is necessary.

Finally, further research should explore whether the sense of urgency for this information will increase if the concept of quality of care becomes more meaningful and patients start to realise that quality and outcomes of care do vary for both treatment and hospital factors.

### Limitations

One strength of our study is the recruitment of respondents in three types of hospitals. Consumer characteristics differed somewhat among the three settings (Table 1), which confirmed the validity of our decision to include the three types. One limitation of our study is that only a minority reported searching for CPI, so we could not make precise estimations, as is reflected in the large confidence intervals in Table 2. We would have preferred a more balanced dataset for better data modelling. Another limitation is the self-reporting of our main outcome, which may have introduced a recall bias. However, we limited the time between selecting a hospital and completing the questionnaire by giving it to the participants at their appointments with a nurse or anaesthetist before surgery. This tactic minimized the time lag between choosing a hospital choice and the date of surgery.

## Conclusions

CPI makes the variation in quality of care between hospitals transparent. This study shows that the number of people who report having searched for CPI is still limited, but may increase if patients become more aware of the quality-of-care variation of hospitals. However, this will be difficult to achieve because people who feel no need for more information – e.g. based on a lack of expected differences in quality of care – do not search for CPI. Awareness as a prerequisite for the use of CPI should not be limited to having knowledge about the existence of CPI and where to find it; awareness should also extend to the quality-of-care variation of hospitals.

## Abbreviations

CI: Confidence interval
CPI: Comparative performance information
GP: General practitioner
OR: Odds ratio
PROMs: Patient-reported outcome measures
